# Toxin Dynamics and Genomic Diversity in *Pyrodinium bahamense* Populations from the Indian River Lagoon, Florida

**DOI:** 10.3390/toxins18100423

**Published:** 2026-09-30

**Authors:** Kathleen D. Cusick, Jackie Palau, Malcolm N. McFarland, Edith A. Widder

**Affiliations:** 1 Harbor Branch Oceanographic Institute, Florida Atlantic University, 5600 US 1 N, Fort Pierce, FL 34946, USA; jmetz2015@fau.edu (J.P.); mmcfarland@fau.edu (M.N.M.); 2 Ocean Research & Conservation Association, Vero Beach, FL 32960, USA; ewidder@teamorca.org

**Keywords:** saxitoxin, bioluminescence, *Pyrodinium bahamense*, harmful algal bloom, copy number variation

## Abstract

*Pyrodinium bahamense* is a saxitoxin-producing bioluminescent dinoflagellate that forms annual recurring blooms in the Indian River Lagoon (IRL), FL. Little information exists as to the extent of bloom toxicity. This study examined toxin dynamics in *P. bahamense* IRL field populations from two sites in the northern IRL (NIRL). Additionally, genomic diversity and transcription were assessed using quantitative molecular assays targeting key functional genes in *P. bahamense* ecology: *sxtA4* (toxin biosynthesis), *rbcL* (photosynthesis), and *lcf* (bioluminescence). A positive correlation was found between *sxtA4* transcript copy number and toxicity, mirroring the trend recorded in previous seasons and indicating that *sxtA4* transcripts can serve as a molecular proxy for bloom toxicity. Lab experiments with 32 strains isolated from the NIRL showed a large variation in copy number for both *sxtA4* (42–318 copies cell^−1,^ dependent on strain) and *rbcL* (1385 to 4911 copies cell^−1^). An *lcf* qPCR assay developed here showed substantial variation among isolates as well. These results are indicative of the underlying diversity in field populations, as genome copy number variation is a major driver of genome evolution and diversity. A negative correlation was found between cell abundance and toxin quota in field populations. Growth and toxin quota were also found to have a negative correlation in the 32 isolates. Based on these collective lab and field results, a model is presented in which *P. bahamense* populations utilize both their toxicity and bioluminescence in bloom development and persistence.

## 1. Introduction

A long-held belief has been that all cells in a dinoflagellate bloom are genetically identical; however, the use of molecular tools in dinoflagellate population ecology studies has identified that dinoflagellates are not monoclonal, even in a monospecific bloom [1,2]. Great phenotypic and genetic diversity occurs within monospecific harmful algal bloom (HAB) populations and/or blooms. Typing with microsatellite markers found high levels of genetic diversity in, for example, toxic *Alexandrium* species from the same geographic area in Cape Cod, Massachusetts; this was also the case with the centric diatom *Ditylum brightwellii* from Puget Sound, Washington [1,3,4]. Among different HAB species, intra-specific variability has been demonstrated for traits including toxin production, growth rate, and motility, among strains isolated from the same water sample and/or geographic location [2,3,5,6]. This clonal diversity results in differences in genome size [7], morphology [8], and growth rate [9]. Population genetic studies with *Alexandrium* revealed differences in toxin production in genetically similar populations, ascribed to natural populations made up of multiple strains that differ in both toxin content and profiles [3,6].

Diversity at the genetic level can occur via genome copy number variation. Copy number variation (CNV), arising from insertion, loss (deletion), or gain (duplication) of bases ranging in size from 50 bp to several kilobases, is a major driver of genome sequence diversity [10]. CNV is viewed as a significant evolutionary process as it can impact the expression of key phenotypic traits [11,12,13,14]. CNVs typically encompass genes that result in different levels of expression, making them key sources of phenotypic variation [10]. Additionally, extensive gene duplication, which has been shown to be a defining characteristic in dinoflagellate genomes, leads to this variation in gene copy number. In general, dinoflagellates can have up to 100s–1000s of copies of the same gene [15,16]. It is important to have an understanding of CNV when studying the ecology and evolution of HABs, as this may result in increased expression and dosage of ecologically relevant genes, thus providing a potential selective advantage. This is especially critical when employing meta-barcoding, qPCR, and metagenomics [17], methods that are being increasingly employed in HAB studies [18,19,20,21].

Saxitoxin (STX) is a potent neurotoxin produced by several species of marine dinoflagellates (*Alexandrium* sp., *Gymnodinium catenatum*, and *Pyrodinium bahamense*) [22]. SxtA is the first enzyme in the dinoflagellate STX biosynthetic pathway; it is a unique polyketide synthase made up of four catalytic domains, coded for by the genes *sxtA1*, *sxtA2*, *sxtA3*, and *sxtA4* [23,24]. Two different forms of SxtA occur: one with all four domains and one lacking sxtA4, and thus, the SxtA4 domain [23]. The N-terminal portion of SxtA is coded for by *sxtA1*; this gene has been found in both toxic and non-toxic strains of toxic species and non-toxic species from other genera [25,26,27]. Biochemical, molecular, and genetic data show that *sxtA4* is specific and essential for toxin synthesis [23,25,28,29,30,31,32]. With the identification of the core STX biosynthesis genes in dinoflagellates, the underpinning genetic mechanisms contributing to differences in toxin content and profiles can be examined directly using molecular tools targeting the STX biosynthetic cluster. Studies on multiple toxic *Alexandrium* species and strains have identified both inter- and intraspecies *sxtA4* CNV. Results differ as to the impact of CNV on toxin production, illustrating the impact of genomic diversity on phenotypic diversity [33,34,35].

*Pyrodinium bahamense* is an STX-producing, bioluminescent dinoflagellate that forms annual recurring blooms in the northern Indian River Lagoon, FL (NIRL). It causes the human illnesses paralytic shellfish poisoning (PSP) and STX pufferfish poisoning (SPFP) [36]. *P. bahamense* has bloomed in the Indian River Lagoon (IRL), along the east coast of Florida, for years with no record of STX production. In the mid-2000s, STX was identified in *P. bahamense* populations in Florida [36,37]; this was the first documentation of STX production in these populations. No molecular data exist on *P. bahamense* populations from this time.

This study sought to identify the underlying genetic and phenotypic diversity within *P. bahamense* blooms through a combination of field analysis and strains isolated from the NIRL. To expand on our field analysis, we examined growth rate, toxin content, and genomic CNV in 32 isolates from various sites in the NIRL. The main aims of this study were (1) to examine toxin quota dynamics in relation to cell abundance and (2) to examine genomic diversity within *P. bahamense* populations via genome copy CNV in 32 isolates from the NIRL. Three genes with ecological relevance were quantified: *sxtA4* (STX biosynthesis), *rbcL* (coding for the large subunit of RuBisCo, a key enzyme in photosynthesis), and *lcf* (coding for luciferase, the key enzyme in bioluminescence). This provided a real-time snapshot of bloom genetics and toxicity as it relates to cell abundance while also examining the molecular and physiological underpinnings at the strain level.

## 2. Results

### 2.1. Cell Abundance and Toxicity Between DB and TM

**Figure 1:**
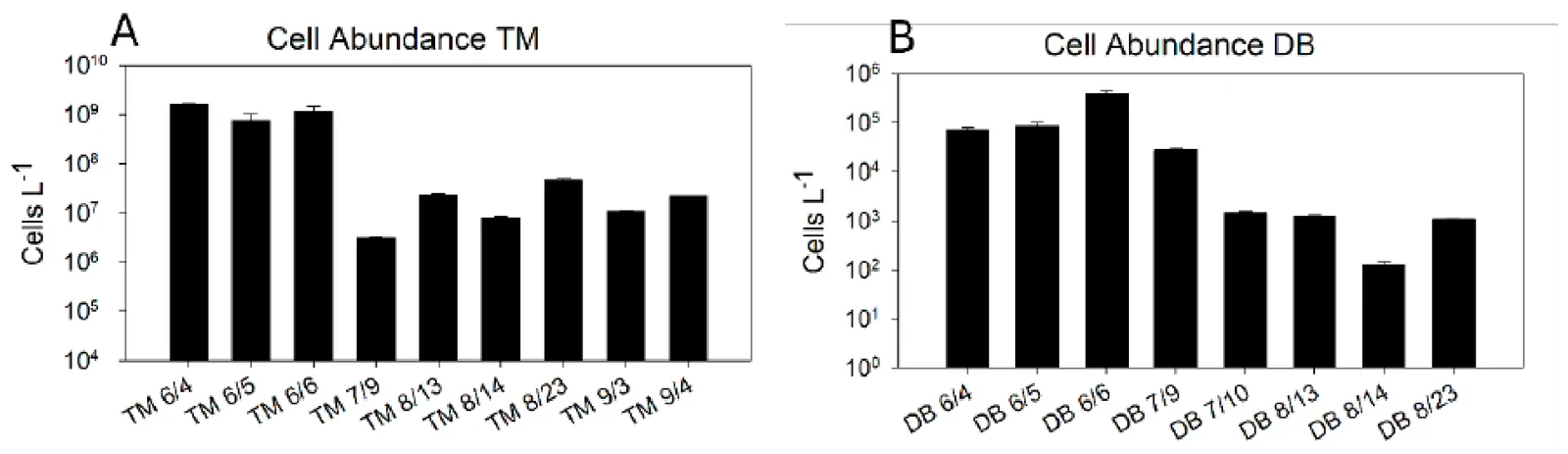
(**A**) Cell abundance (as cells L^−1^) at TM over the course of the 2024 samplings. (**B**) Cell abundance at DB over the course of the 2024 samplings.

**Figure 2:**
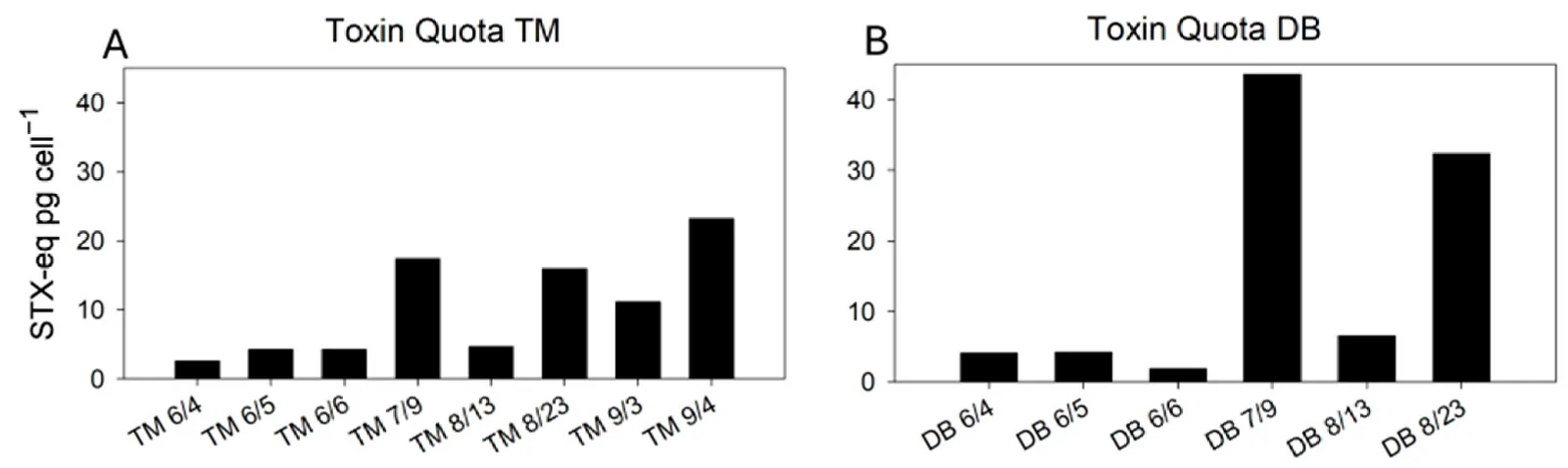
(**A**) Toxin quota per cell (as pg STX-eq cell^−1^) at TM. (**B**) Toxin quota per cell (as pg STX-eq cell^−1^) at DB. There was no significant difference in toxin quota between TM and DB populations, both collectively over the season and when compared among months (*t*-test, no significant difference). Toxin quota at TM ranged from 2.6 to 23.2 pg cell^−1^, with a collective average of 10.4 (±7.7) (Figure 2A). Toxin quota at DB ranged from 1.86 to 43.6 pg cell^−1^, with a cumulative average of 15.5 (SD = 17.9) (Figure 2B). As there was no significant difference in either cell abundance or toxin quota per cell between the two sites, the two were considered together for all further analyses.

There was no significant difference in cell abundance between samples collected at Titusville Marina (TM) and samples collected at Diamond Bay (DB) (Mann–Whitney Rank Sum). Cell abundance at TM ranged from 306 to 163,000 cells L^−1^, with a collective average of 42,500 cells L^−1^ (Figure 1A). Cell abundance at DM ranged from 123 to 3,967,000 cells L^−1^, with a cumulative average of 73,700 cells L^−1^ (Figure 1B). Toxin quota (as STX -eq pg cell-1) at TM ranged from 2.6–23.2, with a collective average of 10.4 (±7.7) (Figure 2A). Toxin quota at DB ranged from 1.86–43.6, with a cumulative average of 15.5(SD = 17.9) (Figure 2B). There was no significant difference in toxin quota between TM and DB populations, both collectively over the season and when compared among months(*t*-test, no significant difference). As there was no significant difference in either cell abundance or toxin quota per cell between the two sites, the two were considered together for all further analyses.

**Figure 3:**
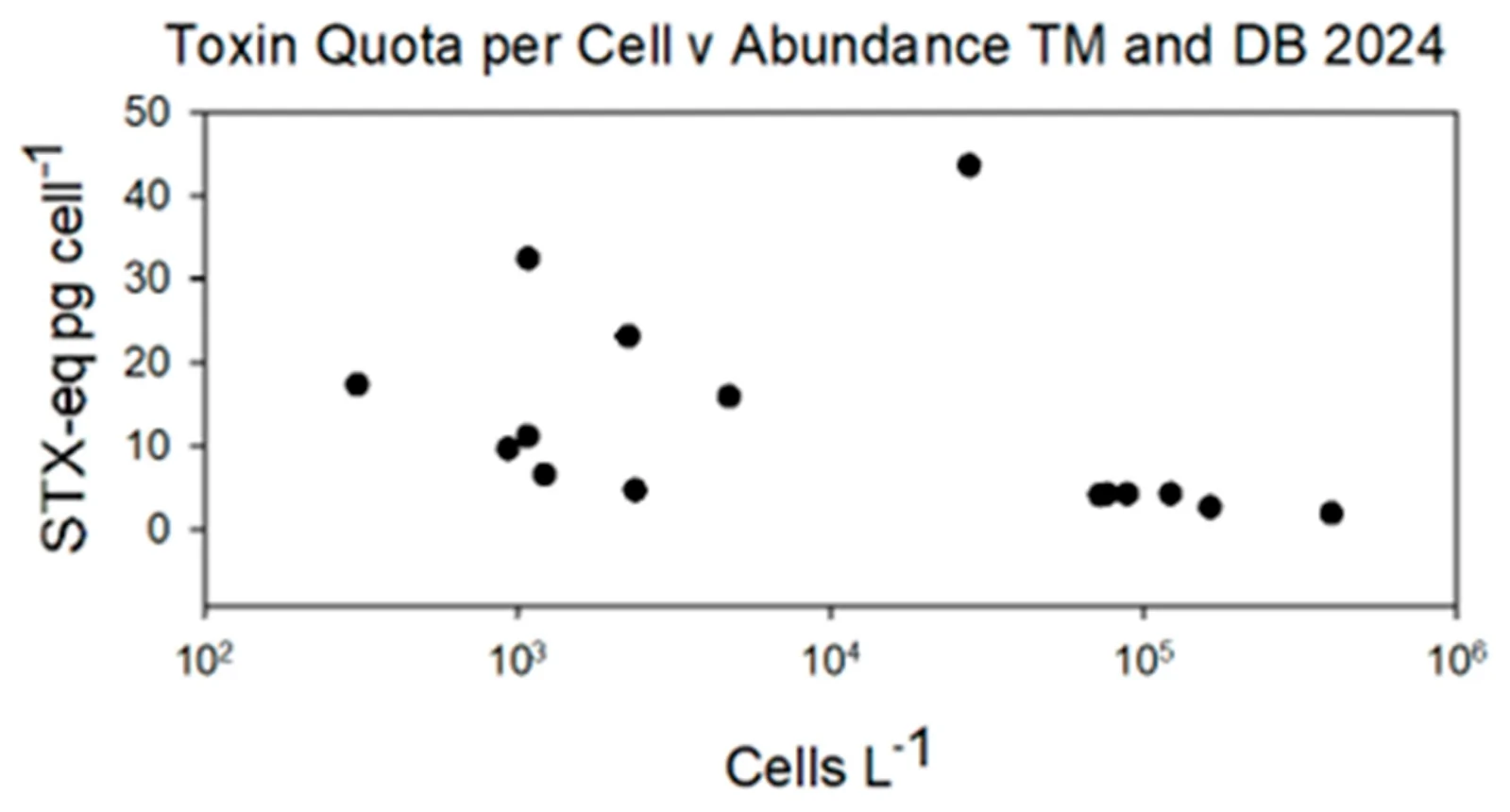
Spearman correlation analysis (Spearman correlation coefficient = −0.86, *p* = 0.0000002) between toxin quota per cell (as pg STX-eq cell^−1^) and cell abundance among cumulative sites and dates (TM and DB, *n* = 15). The x-axis is on a log^10^ scale.

A very strong (Spearman correlation coefficient = −0.86) statistically significant (*p* = 0.0000002) negative correlation was found between the toxin quota per cell (as pg STX-eq cell^−1^) and cell abundance at the combined TM and DB sites (*n* = 15; this is the total number of samples for which there were both cell abundance and toxin measurements) over the 2024 season (Figure 3).

### 2.2. Gene and Transcript Abundance of sxtA4 and rbcL in P. bahamense Field Populations

**Figure 4:**
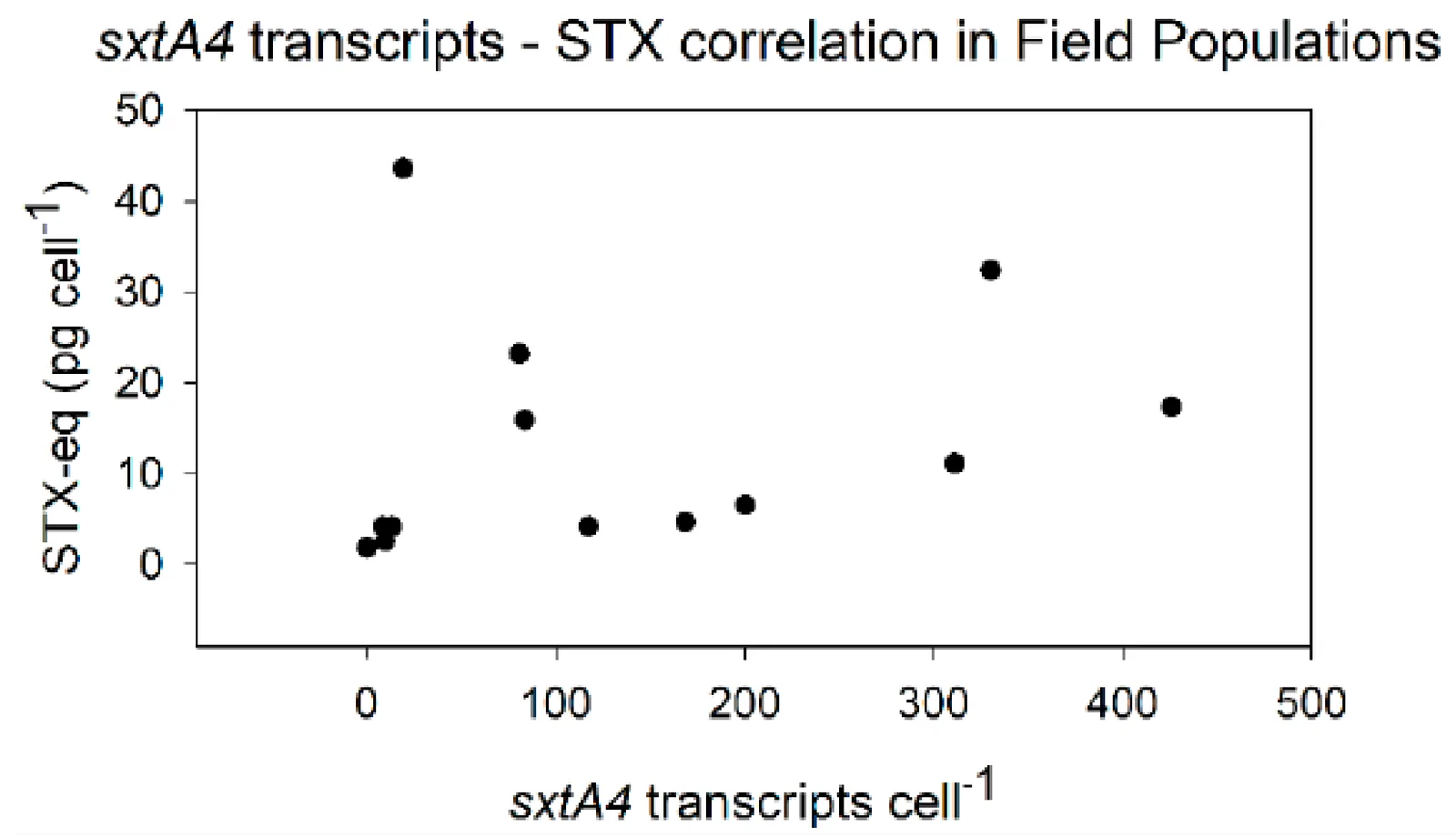
Spearman correlation analysis between *sxtA4* transcripts and STX quota per cell (as pg STX-eq cell^−1^) (Spearman correlation coefficient 0.67, *p* = 0.008, *n* = 14). The *sxtA4* qRT-PCR assay was used to quantify *sxtA4* transcript abundance, which was normalized to cell abundance of the respective sample.

*SxtA4* transcript abundance showed a strong positive correlation with toxicity (Spearman correlation coefficient 0.67, *p* = 0.008, *n* = 14, where *n* is the total number of samples for which there were sxtA4 transcript and toxin measurements) (Figure 4). This followed the trend recorded in previous seasons, in which *sxtA4* transcripts also showed a positive correlation with *P. bahamense* toxicity from multiple sites within the NIRL. Unlike *sxtA4* transcripts, the sxtA4 gene copy number did not correlate with toxicity (Spearman correlation coefficient 0.26, *p* = 0.36, Figure S1). These data demonstrate that *sxtA4* transcripts can serve as a molecular proxy for active toxin synthesis in *P. bahamense* populations. Additionally, this indicates that transcripts, rather than gene copies, better inform as to the toxicity of a population.

**Figure 5:**
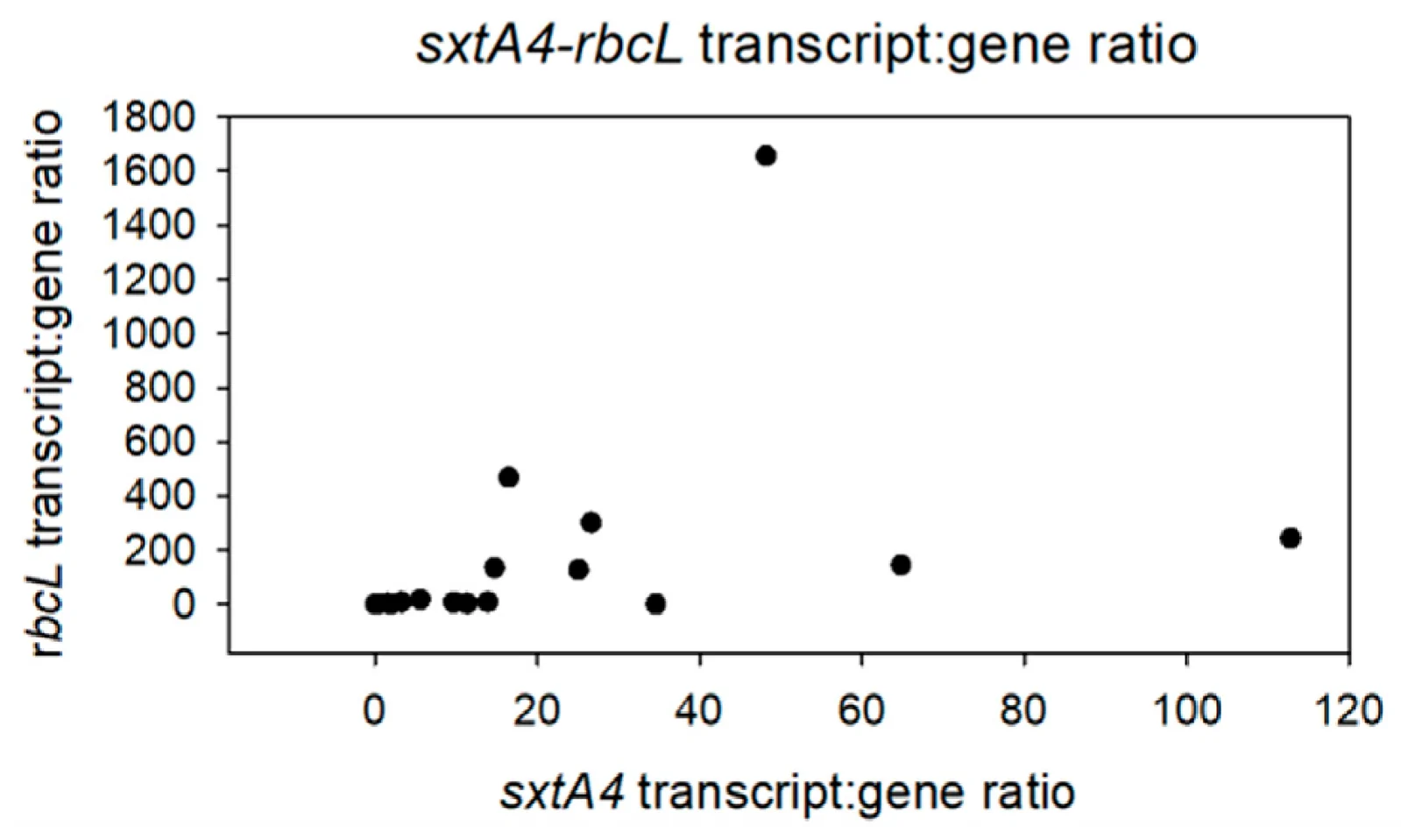
Spearman correlation analysis of the *sxtA-rbcL* transcript: gene ratio (Spearman correlation coefficient = 0.664, *p* = 0.003, *n* = 17). *SxtA4* and *rbcL* transcript and gene copies were quantified using qPCR assays described previously and normalized to cell abundance.

*P. bahamense* is a photosynthetic dinoflagellate. As a gauge of toxin synthesis relative to overall bloom activity as carbon fixation, we measured *rbcL* transcripts and gene copies. The *rbcL* transcript:gene ratio was then correlated with the *sxtA4* transcript: gene ratio as a qualitative assessment of toxin synthesis (*sxtA4*) coupled with bloom metabolism (photosynthesis, as *rbcL*). A statistically significant positive correlation was found between the two (Spearman correlation coefficient = 0.664, *p* = 0.003, *n* = 17), indicating that toxin synthesis occurred in photosynthetically active populations (Figure 5).

### 2.3. Development of an lcf Quantitative PCR Assay

A qPCR assay was developed to measure the *lcf* copy number in the 32 *P. bahamense* isolates. As stated in the Section 4, two *lcf* variants were consistently obtained from *P. bahamense* cells from the NIRL in a previous study. The primers of the qPCR assay developed here target conserved regions of both variants. This was confirmed by sequencing approx. 15 qPCR products from the specificity tests (described below) and the 32 isolates.

The *lcf* qPCR assay was tested on multiple *P. bahamense* strains, and all strains yielded a single peak via melt curve analysis (Figure S2). The primers were also tested on multiple species of bioluminescent dinoflagellates, including *Pyrocystis lunula*, which has the most similar *lcf* sequence to *P. bahamense*, *Pyrocystis fusiformis*, and *Alexandrium monilatum*, which has also been reported in the NIRL; non-bioluminescent dinoflagellates (*Karlodinum veneficum* and *Karenia brevis*) were also tested to account for any non-specific binding. No amplification occurred with any of the species. As our goal was to determine the copy number in *P.bahamense* lab isolates, the most important parameter was specificity among all *P. bahamense* isolates, defined as the ability to amplify *lcf* and yield a single peak. This was achieved (Figure S2). The assay characteristics were slope = −3.078, y-intercept = 39.21, r2 = 0.9584, and PCR efficiency = 111%, and it was able to reliably and consistently detect down to 10 copies (the final point of the eight-point standard curve) (Figure S3).

The primers bind to all three domains and are not specific to a certain one (i.e., D1, D2, D3). This was determined by amplifying the conserved catalytic domain region from multiple isolates (i.e., PCRs yielding a 370 bp product) and sequencing these products. 

The sequences targeted by the primers are conserved among isolates and domains. Heterozygosities in the electropherograms of the cleaned 370 bp PCR products indicates the different domains. Primers do not target these regions of base differences.

### 2.4. Correlation Between Growth Rate and Toxicity in Lab Isolates

**Figure 6:**
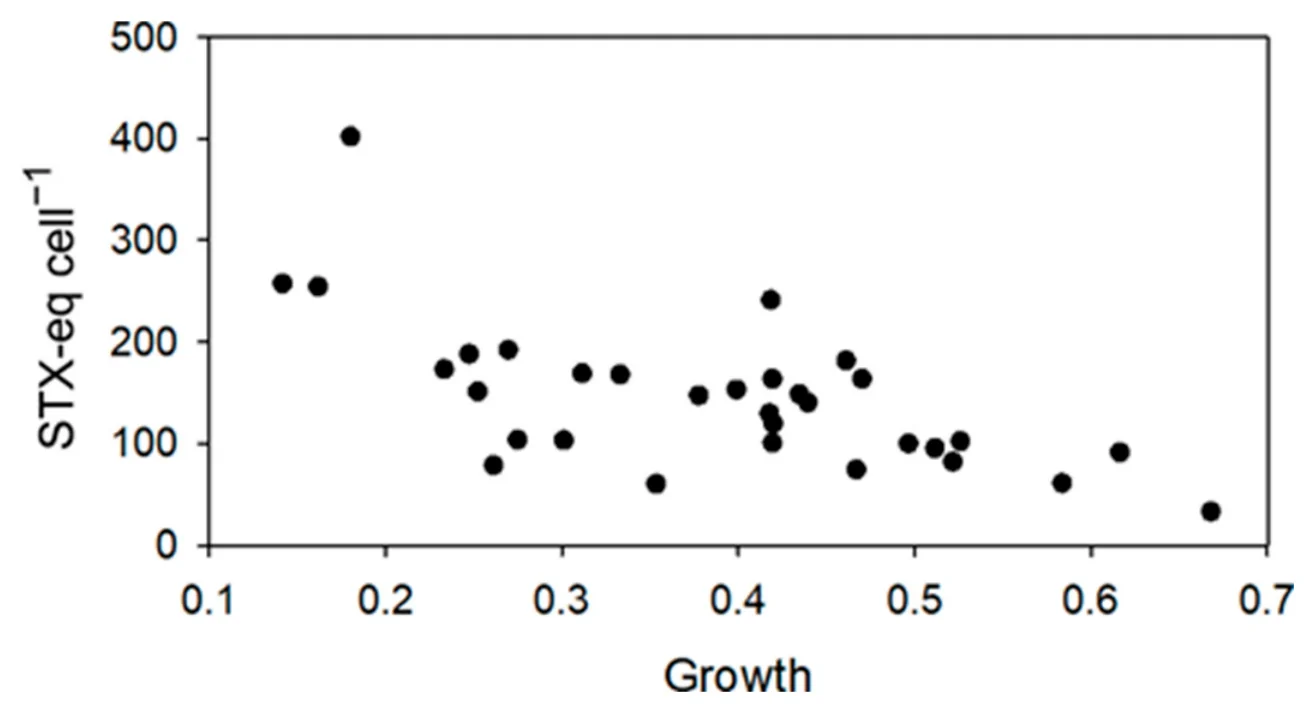
Correlation analysis between STX quota per cell (as measured via the PSP ELISA kit) and growth rate (as cell division rate per day) in 32 *P. bahamense* strains isolated from the NIRL (Pearson product correlation = −0.65, *p* = 0.000006). Subsamples from mid-exponential clonal cultures (1 mL) were diluted 1000× prior to analysis with the ELISA kit; the dilution factor was accounted for when plotting the data.

A strong negative correlation was found between the cell division rate and STX (as fmol cell^−1^) among strains (Pearson product correlation = −0.65, *p* = 0.000006, Figure 6), indicating that strains that produced higher levels of toxins grew more slowly than those producing less.

### 2.5. Correlation of sxtA4 Gene Copy Number with Toxicity in Lab Isolates

**Figure 7:**
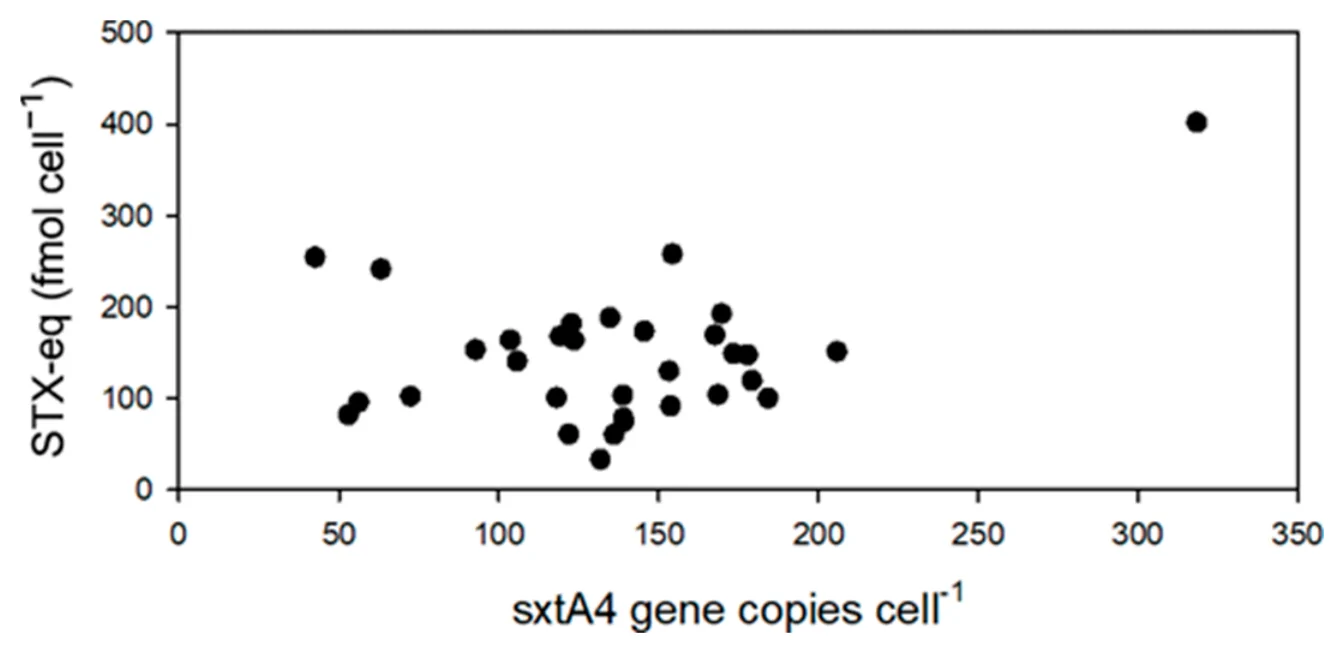
Pearson product correlation analysis between *sxtA4* gene copy number and toxin production (as STX-eq fmol cell^−1^) among 32 IRL *P. bahamense* isolates showed no relationship. *SxtA4* gene copy number was normalized to a per-cell basis for each isolate.

**Figure 8:**
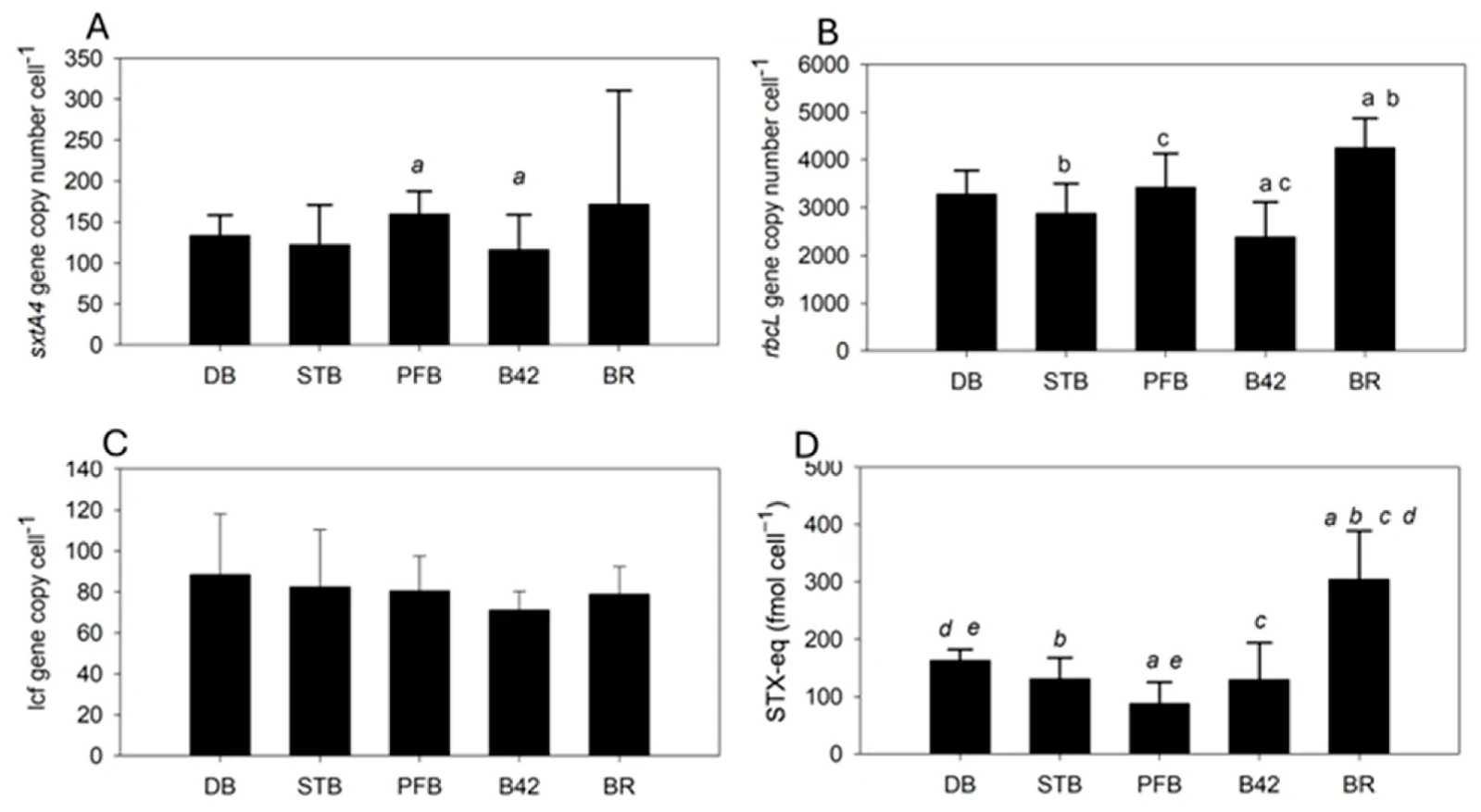
Average gene copy number and STX quota per cell (as fmol cell^−1^) among IRL strains based on isolation location. (**A**) *SxtA4* copy number per cell from strains among sites. The average *sxtA4* copy number was determined by taking the *sxtA4* gene copy values for each isolate for the respective site. (**B**) Average *rbcL* copy number per cell from strains among sites, determined as for *sxtA4*. (**C**) *lcf* gene copy number per cell among sites. (**D**) STX concentration (as fmol cell^−1^) among sites. Lowercase letters for each panel indicate statistically significant differences (*p* < 0.05) between those sites as described in the text. The number of isolates from each site is as follows: DB *n* = 7; STB *n* = 8; PFB *n* = 7; B42 *n* = 7; BR *n* = 3.

Our field data showed no correlation between the *sxtA4* gene copy number and toxicity. We wanted to examine this relationship among individual strains, and so we measured these two features in 32 strains (see Table 1 for isolation location and strain nomenclature) isolated from the NIRL. There was no correlation (Pearson product correlation analysis) between the *sxtA4* gene copy number and STX quota per cell (as fmol cell^−1^) (Figure 7) when taking all 32 isolates collectively. When examining the relationship between *sxtA4* gene copy number and toxin quota for strains based on site, a strong statistically significant positive correlation (Pearson product correlation coefficient = 0.934, *p* = 0.002) was found for PFB strains; there was no correlation (positive or negative) among strains from the other four sites. This corroborates the findings with field populations, in which the *sxtA4* gene copy number did not correlate with STX production (Figure S1). The STX values (as fmol cell^−1^) measured among isolates based on location were as follows: DB = 163.5 ± 18; ST = 131 ± 37; PF= 88 ± 37; B42 = 130 ± 64; BR = 304 ± 84 (Figure 8D). Pairwise multiple comparisons (Holm–Sidak method) showed that STX values were statistically significantly different between the following: BR and PFB (*p* < 0.001), BR and ST (*p* < 0.001), BR and B42 (*p* < 0.001), BR and DB (*p* = 0.001), and DB and PFB (*p* = 0.031).

### 2.6. Genome Copy Variation Among Isolates

The average *sxtA4* gene copy number for the 32 NIRL isolates was 136.5 ± 52.7, with a minimum copy number of 42 (BR17-23) and a maximum of 318 (BR17-16) (Table 2). Even when separating isolates by location, substantial variability in copy number remained. The average *sxtA4* gene copy number among isolates from each location was as follows: DB = 134 ±24.7 (*n* = 7); ST = 122 ± 48.5 (*n* = 8); PFB = 160 ± 28 (*n* = 7); B42 = 116 ± 42 (*n* = 7); BR—172 ± 139 (*n* = 3) (Figure 8A). While displaying a great range, a statistically significant difference was found only between sites PFB and B42 (Student’s t, *p* < 0.05). It is worth noting that BR had the greatest *sxtA4* copy number average, but also the greatest range, with one isolate containing only 42 gene copies (Table 2). Overall, these data suggest the underlying genetic diversity within *P. bahamense* populations.

The average *rbcL* copy number among the 32 strains was 3108 ± 805, with a minimum copy number of 1385 (B42B-2) and a maximum of 4911 (PFB-6). The *rbcL* copy numbers among isolates from each site were DB = 3285 ± 491; ST = 2880 ± 626; PF = 3429 ± 707; B42 = 2384 ± 730; BR = 4251 ± 618 (Figure 8B). A one-way ANOVA performed among the five locations showed a statistically significant difference (*p* = 0.002). When comparing *rbcL* gene copy numbers among isolates based on location, pairwise multiple comparisons (Holm–Sidak method) found a statistically significant difference between BR and B42 (*p* = 0.003), BR and STB (*p* = 0.035), and PFB and B42 (*p* = 0.041) (Figure 8B). The wide range in copy numbers recorded for *sxtA4* did not occur for *rbcL* among BR isolates, with an SD consistent with that seen among isolates at the other locations (Table 1).

The average copy number of *lcf* among all isolates, when normalizing for amplification of all three domains, was 83.5 ± 20.9, ranging from 39.5 (ST-4) to 132.6 (DBB-16) (Table 2). Among isolates based on site, average copy numbers were as follows: DB = 96.5 ± 24.8; ST = 86.7 ± 29.5; PFB = 80.8 ± 9.31; B42 = 73.7 ± 10.1; BR = 74.1 ± 14.4 (Figure 8C). A one-way ANOVA performed among sites did not identify a significant difference in copy numbers.

*SxtA4*, *rbcL*, and *lcf* are key genes in the *P. bahamense* lifestyle; collectively, the CNV among NIRL strains suggests genomic diversity within natural field populations. Even those from the same geographic areas within the NIRL show substantial variation (Table 1). The difference in gene copy numbers among strains reflects what may be occurring within a bloom: a mix of strains, with varying degrees of toxicity.

## 3. Discussion

Our field data show that there is a strong, statistically significant negative correlation between cell abundance and toxin quota per cell in spatially and temporally separate *P. bahamense* populations; this is the same trend recorded over previous seasons [38]. The inverse relationship between toxin quota per cell and cell abundance in field populations of *P. bahamense* also occurred in lab isolates. Here, strains that produced higher levels of STX grew more slowly than those that produced less (Table 1). These data suggest a scenario as to how strain diversity, and thus toxin biosynthesis, may influence bloom development. Dinoflagellates typically exist as unicellular, vegetative cells; thus, their net growth rate is a balance of the cell division rate and mortality rate due to predators [40]. Copepods, one of the main predators of dinoflagellates, and their chemical cues (copepodamides) have been shown to induce toxin production in dinoflagellates [41,42]. However, chemical defenses are costly, resulting in reduced physiological efficiency, manifested as mortality or slowed growth [43]. A range of results exist in the literature as to the costs of toxin production. Costs are influenced by multiple factors, including predator chemical cues, light, and nutrients [44]. Most of these data are derived from lab studies. Ecological modeling predicts that the cost of toxin production can result in a >20% decrease in the cell division rate [45]. Leading up to bloom initiation, factors such as competition with co-occurring phytoplankton species and high grazing pressure may select for strains with increased toxin production. However, during bloom development, strains that can grow more quickly benefit the population as a whole.

CNV, arising from insertion, loss (deletion), or gain (duplication) of bases ranging in size from 50 bp to several kilobases, is a major driver of genome sequence diversity [10]. CNV is viewed as a significant evolutionary process as it can impact the expression of key phenotypic traits [11,12,13,14]. Additionally, extensive gene duplication, which has been shown to be a defining characteristic in dinoflagellate genomes, leads to variation in gene copy numbers. In general, dinoflagellates can have up to 100s–1000s of copies of the same gene [15,16]. *Sxt* gene duplication and loss seem common [25] and, as is the trend with dinoflagellate genes, are present in numerous copies due to processes such as the recycling of processed cDNAs back into the genome through reverse transcription, retroposition [46,47,48], and potentially environmental stress [49].

A recent study on *sxtA4* genome CNV in *Alexandrium* species found high variability among species and, to a lesser extent, among strains: *sxtA4* genome CNV in *A. pacificum* ranged from ca 8 to 610 copies per cell; *A. minutum* and *A. australiense*, 0 to ca 9 copies per cell; and *A. catenella*, ca 20 to 190 copies per cell [17]. The collective trend in this recent study found the *sxtA4* copy number to correlate with the amount of total paralytic shellfish toxins, indicating a gene dosage effect modulating toxin production [17]. However, previous studies with multiple species and strains of STX-producing *Alexandrium* vary as to the correlation between the *sxtA4* gene copy number and toxicity. In 15 strains of *A. minutum*, *sxtA4* strongly correlated with toxin content, while no correlation was found between copy number and toxin content in three strains of *A. ostenfeldii* [33,34]; there was also no difference in genome copy number in *A. catenella* between toxic and non-toxic strains [35]. Unlike some *Alexandrium* species, in which genome CNV was significantly related to toxicity (as ng cell^−1^ PSTs), thus suggesting a gene dosage effect modulating PST production, this does not appear to be the case for *P. bahamense*.

When considering mechanisms enabling *P. bahamense* bloom development and persistence, the other key phenotype that should be considered is its bioluminescence. Some of the most frequent and prominent toxic dinoflagellate blooms occur in species that are also bioluminescent, yet the two traits are rarely studied together in HAB development [50]. Bioluminescence in dinoflagellates also functions as a defense from predators [50]. It has been shown to reduce grazing by zooplankton predators [51,52], with copepods consuming fewer dinoflagellates in their bioluminescent night phase compared to their non-bioluminescent day phase [52,53]. Copepod chemical cues (copepodamides) are documented to increase the total bioluminescence capacity in both *A. tamarense* and *L. polyedra* [54], and this copepodamide-induced bioluminescence causes a shift in copepod feeding preference [55]. As the doubling rate of dinoflagellates is one third that of co-occurring diatoms [56], selective feeding by grazers on non-luminescent prey gives an advantage to the bioluminescent dinoflagellates. How bioluminescence functions as a defense seems to differ between toxic and non-toxic species [50]. In general, smaller (~20–40 uM) cells with a less intense flash (3.1 × 10^7^–1.2 × 10^8^ photons cell^−1^), such as *L. polyedra* (yessotoxin) and *P. bahamense*, are toxic, while larger cells with a more intense flash (3.7 × 10^10^–6.5 × 10^11^ photons cell^−1^), such as *Pyrocystis* spp., are not [50]. A primary hypothesis is that the flash serves as a “burglar alarm” to attract secondary predators of the dinoflagellate’s predator [57,58,59,60]. Since many bloom-forming species are also toxic, an alternative hypothesis is that the flash serves as an aposematic warning in toxic species, signaling unpalatability to grazers [50]. Bioluminescence as an aposematic signal was initially suggested several decades ago [61] and has since been shown to function as an aposematic warning in firefly larvae [62,63,64], millipedes [65], and brittle stars [66,67]. In a dark environment such as the ocean at night, the light is the equivalent of aposematic coloration, warning potential predators of toxicity. Previous work with toxic bioluminescent dinoflagellate species indicated it functioned as an aposematic signal at low cell concentrations and a burglar alarm at high [68,69].

An extension to aposematic signaling is Batesian mimicry, in which individuals of a palatable, non-toxic species (the mimic) benefit from reduced predation due to their resemblance to a toxic species that predators avoid [70,71]. Strong support for Batesian mimicry in dinoflagellates has come from experiments in the Widder lab that looked at copepod grazing on toxic and non-toxic bioluminescent dinoflagellates [68]. Automimicry is Batesian mimicry within the same species [43,72]. Our recent field data showing that both *sxtA4-* and *sxtA4+* genotypes occur in *P. bahamense* populations indicate that an automimicry-like process may be occurring during bloom development. With this scenario, strains decrease their toxicity, enabling faster growth, and retain their bioluminescence, relying on the mimicry process as protection from grazing copepods. Future work to test this hypothesis includes cumulative toxin and bioluminescence measurements in multiple *P. bahamense* isolates coupled with growth measurements.

## 4. Materials and Methods

### 4.1. Field Sampling

The Indian River Lagoon, located along Florida’s east coast, is an estuarine system consisting of multiple interconnected basins spanning 156 miles from Ponce de Leon Inlet (Volusia County) to Jupiter Inlet (Palm Beach County). *P. bahamense* forms recurring blooms in the northern IRL, encompassing the northern portion of the Indian River, the Banana River, and the Mosquito Lagoon from approximately May to September, though blooms have been increasing spatially and temporally (Cusick, pers. obs., FWC HAB reports). Field sampling was conducted at two sites between April and September 2024: Diamond Bay (“DB”, 28.3503 N, 80.6131 W) and the Titusville Marina (“TM”, 28.6222 N, 80.8083 W) in the NIRL. In general, the two sites were sampled on the same or similar dates, for 2–3 consecutive days. The following parameters were measured (described in detail below): cell abundance; toxin quota per cell; *sxtA4* and *rbcL* gene copies; *sxtA4* and *rbcL* transcripts.

Near-shore whole water samples were collected as described previously. Briefly, water was collected from the surface to 0.25 m and concentrated by size-fractionating 7.5–20 L through a 30-micron mesh (*P. bahamense* width is on average 40 microns). Biomass was rinsed from the filter into sterile Nalgene bottles using filtered site water. Three biological replicates (i.e., three water samples) were collected for RNA extraction; two biological replicates were collected for DNA extraction; aliquots from these bottles were used for toxin analysis and cell enumeration. Samples were transported at ambient temperature back to the lab. Samples were filtered under low vacuum immediately upon return to the lab. Samples for DNA and RNA extraction were collected onto Nucleopore (1 µm) polycarbonate filters. Filters for RNA extraction were covered in RNALater (Invitrogen, Carlsbad, CA, USA) and incubated for 3–5 min at room temperature prior to storage at −20 °C. Samples for toxin analysis were filtered onto Whatman AE filters and stored at −20 °C until extraction. Samples for cell counts were preserved with 2% glutaraldehyde and stored at 4 °C until enumeration via light microscopy(Nikon Instruments, Melville, NY, USA).

### 4.2. P. bahamense Enumeration

Cell counts were performed as described previously [38]; briefly, 1 mL aliquots were placed onto a Sedgewick Rafter counting chamber, allowed to settle, and counted under standard light microscopy (200–400× magnification based on morphology [73]). Triplicate cell counts were performed for each sample.

### 4.3. STX Analysis of Field Samples

STX was extracted and analyzed using a commercial Saxitoxin (PSP) ELISA kit (Eurofins Abraxis LLC, Warminster, PA, USA) according to the manufacturer’s instructions, as previously described [38]. Briefly, STX (as cells collected on a filter) was extracted with Milli-Q water containing 1% acetic acid (*v*/*v*) and combined with 5 mL of extraction solvent. The combination was freeze-thawed three times. Extracts were clarified by centrifugation at 16,000× *g* for 10 min and passed through a 0.45 µm nylon syringe filter. The supernatant was analyzed in duplicate using the commercial Saxitoxin (PSP) ELISA kit. When needed, appropriate dilutions were applied to fit the standard curve range (0–0.4 ppb). Values were then normalized to cell abundance (cells L^−1^) for that sample, so that values are reported as pg cell^−1^.

### 4.4. Nucleic Acid Extraction Field Samples

For both DNA and RNA extraction, filters were removed from the microfuge tubes using sterile forceps and placed into the appropriate kit-supplied bead tube for lysis. For RNA samples, excess RNALater was removed by pressing the forceps against the filter(s) while in the microfuge tube prior to transfer to the bead tube. DNA was extracted using the DNeasy Plant Pro kit (Qiagen, Germantown, MD, USA). Samples were lysed via three rounds of bead beating using a Genie Vortexer (Scientific Industries, Bohemia, NY, USA)with high-speed agitation for 10 min. Samples were eluted in 100 ul nuclease-free water and stored at −80 °C until use in quantitative PCR. Total RNA was extracted using the RNeasy Power Plant kit (Qiagen) with bead beating for three rounds of 7 m/s for 35 s using an MP Biomedical FastPrep bead beater (MP Biomedicals, LLC, Solon, OH, USA). The samples were chilled on ice for 45 s in between each round. The remaining steps followed the manufacturer’s protocol. The optional on-column DNase digest was performed, with the digestion time extended to 20 min. Samples were eluted in 50 µL RNase-free water and stored at −80 °C until cDNA synthesis. Total RNA concentration and purity were assessed using the Nanodrop 1C (ThermoFisher, Waltham, MA, USA). Total RNA was converted to cDNA using the High-Capacity RNA-to-cDNA kit (Applied Biosystems, Foster City, CA, USA). Each reaction contained 10 µL 2× RT buffer, 1 µL 20× enzyme, and 0.8 ul total RNA and was brought to a final volume of 20 µL with nuclease-free water. No-RT controls were performed to confirm the absence of DNA and consisted of all components except the enzyme. Reactions were performed on the ProFlex thermocycler (Applied Biosystems, Carlsbad, CA, USA) at 37 °C for 60 min, followed by 95 °C for 5 min.

### 4.5. Establishment of Clonal P. bahamense Cultures and Determination of Growth Rates

Thirty-two strains of *P. bahamense* were established from multiple locations in the northern Indian River Lagoon (NIRL). The 32 strains profiled here along with their date and location of isolation are listed in Table 1. Single-cell isolation using a stereo microscope (Nikon Instruments, Melville, NY, USA) and micropipette was performed for surface water samples collected from four sites in the NIRL: Diamond Bay (DB), Beacon 42 (B42), Station 1 (ST), and Platform (PF) in 2017 and 2022. Individual *P. bahamense* cells were washed to remove picoplankton, which can rapidly overgrow cultures, and inoculated into sterile L1 medium using water from the Banana River as a base (autoclaved Banana River water supplemented with L1 nutrients [74], without Na_2_SiO_3_, and filter-sterilized over 0.2 µm PES filters). Cultures exhibiting high picoplankton density (green coloration) were excluded. A total of 32 clonal cultures (3 isolated in 2017 and 29 isolated in 2022) were inoculated at ~500 cells mL^−1^ into 30 mL of BR L1 medium and grown to the mid-exponential phase (~3000–4000 cells mL^−1^). At this stage, subsamples were collected for flow cytometry, toxin production measurements (ELISA), and DNA extraction. The remaining culture was diluted 1:3 with medium and regrown to the mid-exponential phase; this process was repeated three times to generate three independent replicates per culture. Growth of the triplicate cultures of all strains was measured via flow cytometry starting upon inoculation (day 0) and ca every 1–2 days from day three.

### 4.6. DNA Extraction of Clonal Cultures

Subsamples from mid-exponential clonal cultures (10 mL) were filtered through 0.2 μm GV Durapore^®^ membranes, stored at 20 °C, and extracted within 24 h using the Qiagen PowerWater DNA Isolation Kit (Qiagen, Germantown, MD, USA). Filters were incubated in P1 lysis buffer for 10 min at 60 °C to ensure complete cell lysis. Extracted DNA was stored at −80 °C until use in quantitative PCR assays.

### 4.7. Toxin Analysis of Clonal Cultures

The Saxitoxin (PSP) ELISA kit (as stated above in STX Analysis of Field Samples) was used to measure toxin production in the 32 isolates. Subsamples from mid-exponential clonal cultures (1 mL) were diluted 1000× with ultra-pure water and then run with the ELISA kit. A BR L1 medium control was also included to ensure that the growth medium did not contain significant levels of saxitoxin, which could affect the results. The toxin concentrations for *P. bahamense* isolates per cell (STX equivalent[eq]/cell) were calculated using cell concentrations determined by flow cytometry.

### 4.8. Quantitative Reverse Transcription PCR and Quantitative PCR of Field Samples

Quantitative Reverse Transcription PCR (qRT-PCR) assays were previously developed for *P. bahamense* [38]. These assays targeted *sxtA4* and *rbcL*. As samples were collected during the day, and *P. bahamense* is photosynthetic, the *rbcL* assay served as a proxy for gauging carbon fixation and thus general transcriptional activity of the population. Assay design and optimization were previously reported [38]. Both assays utilized the PowerUp SYBR Green master mix (Applied Biosystems, Foster City, CA, USA) and were performed on the QuantStudio 6 Real-Time PCR System with the fast 96-well block format. Each reaction contained 10 μL of 2× PowerUp SYBR Green master mix, 300 nM of each forward and reverse primer, and 2 μL of template cDNA, brought to a final volume of 20 μL with nuclease-free water. No-RT controls consisted of all components except the enzyme. The following protocol was used for all reactions: an initial 20 s incubation at 95 °C, followed by 40 cycles of 95 °C for 1 s and 60 °C for 20 s, followed by a melt curve analysis of 95 °C for 15 s, 60 °C for 1 min, and 95 °C for 15 s to determine product specificity. All reactions were performed in duplicate. Transcript abundance for each target was determined via absolute quantification using eight-point standard curves as described previously [38]. Briefly, standard curves were constructed from purified PCR products of the nearly full-length *sxtA4* and *rbcL* genes amplified from a lab isolate. The number of copies per µL was calculated using the equation X g µL^−1^ DNA/[PCR amplicon × 660]) × 6.022 × 10^23^. The PCR product stock solution was diluted in nuclease-free water to a working concentration of 5 × 10^8^ copies per µL. Standard curves spanning eight orders of magnitude from 5 × 10^7^ to 5 × 10^−1^ copies per µL were prepared via serial dilutions of the stock solutions. In total, 2 µL of each dilution was used per reaction, yielding standard curves that ranged from 1 × 10^1^ to 1 × 10^8^ copies. Data were recorded using the Applied Biosystems QuantStudio 6 software as total transcripts in the 20 µL reaction; these numbers were then normalized to transcripts per cell based on cell abundance of the sample and accounting for the volume of RNA extract, cDNA synthesis, and qPCR volumes. These same assays were also used to measure the gene copies of each target. The assays utilized the same reaction set-up and SYBR Green chemistry, but with 2 µL of the DNA extract as a template rather than cDNA. Gene copies were calculated using absolute quantification with eight-point standard curves and normalized to copies per cell based on cell abundance and accounting for the volume of DNA extract and qPCR volume.

### 4.9. Development of lcf Quantitative PCR Assay

Bioluminescence is the generation of light by living organisms via a biological reaction. The dinoflagellate bioluminescent reaction consists of the substrate, luciferin, which is oxidized by molecular oxygen via the luciferase (LCF) enzyme and manifested as a flash of blue light (=peak emission is 472–475 nm) [75,76,77]. Sequencing of multiple photosynthetic dinoflagellate species showed LCF codes for a single polypeptide that contains an N-terminal region of unknown function followed by three homologous domains (“D1,” “D2,” “D3”), each with catalytic activity [78,79]. The individual domains among species (i.e., D1 *Pyrocystis fusiformis*, D1 *P.noctiluca*) are more similar than among the three different domains of the same species (i.e., D1, D2, D3 of *P. fusiformis*). There currently exists no publicly available full-length *lcf* genomic sequence for *P. bahamense*, and all fully sequenced LCFs (from multiple photosynthetic dinoflagellates [78]) are derived from cDNA sequences. Previous work with single cells of *P. bahamense* collected from multiple locations around the NIRL showed that two different *lcf* variants were consistently recovered [39]. Sequences were obtained using degenerate primers that amplified a ca 370 bp region of the conserved catalytic domain [80]. These variants formed two distinct clusters, which were defined by a set of core amino acid differences resulting from non-synonymous substitutions (representative GenBank accession numbers KX377135, hereafter referred to as “lcf22”, and KX377141, hereafter referred to as “lcf23” for DNA sequences).

A quantitative PCR assay for measuring *lcf* was developed based on conserved catalytic domain sequences. Primers were designed based on *P. bahamense* sequences from field samples and clonal isolates (GenBank accession numbers KX377133.1-KX377170.1) [39]. Sequences were aligned in MEGA version 7 [81] using ClustalW. Primers were manually designed and analyzed for parameters such as annealing temperature, secondary structures, and potential dimerization using the Oligonucleotide Properties Calculator (V3.19) (URL accessed on 30 March 2024, http://biotools.nubic.northwestern.edu/OligoCalc.html) and IDT OligoAnalyzer Tool URL accessed on 20 March 2024, https://www.idtdna.com/pages/tools/oligoanalyzer). Multiple primers were designed that targeted conserved regions of the 370 bp sequence. Primer concentrations of 200, 400, and 600 nm (as final concentrations) were evaluated with temperatures of 58, 60, and 62 °C using the PowerUp Fast SYBR Green master mix (Applied Biosystem, Foster City, CA). The optimized annealing/extension temperature was found to be 60 °C, with a final primer concentration of 600 nM. The following primer set was selected: Lcf54F, 5′-AG-GCCATCGAGGCGGGC and Lcf154R, 5′-CAGTGCAGACATCGCCAAAC (Table 2). The assay was optimized using genomic DNA from multiple *P. bahamense* isolates. Assay specificity was assessed by performing qPCRs on: 1. Multiple *P. bahamense* isolates. 2. The bioluminescent dinoflagellates *Pyrocystis fusiformis*, *P. lunula*, and *Alexandrium monilatum*, which have the most similar LCFs to *P. bahamense* and can co-occur with *P. bahamense* in the IRL. 3. The non-bioluminescent dinoflagellates *Karlodinium veneficum* and *Karenia brevis*. The PCR efficiency and sensitivity were determined by creating an eight-point standard curve from the 120 bp purified PCR product of the target region of the *lcf* gene amplified from the genomic DNA of *P. bahamense*. Serial 10-fold dilutions were used to construct a standard curve spanning eight orders of magnitude from 1 × 10^8^ to 1 × 10^1^ copies.

### 4.10. Quantitative PCR of Lab Isolates

The *sxtA4*, *rbcl*, and *lcf* assays were applied to the 32 strains of *P. bahamense* isolated from the NIRL. Assays were performed as described for the field samples, using 2 ul of genomic DNA from each biological replicate. Thus, for each assay, duplicate qPCRs were performed on three biological replicates of each strain. Gene copies were normalized to cell abundance, so that data were recorded as gene copies per cell.

## Figures and Tables

**Table 1:** List of all strains, dates and locations of isolation, and *sxtA4*, *rbcL*, *lcf*, STX (as fmol cell^−1^), and growth measurements.

Isolate	Date Isolated	Location	Latitude	Longitude	*sxtA4*	*rbcL*	*lcf*	STX	Growth
DBB-16	08/26/22	Diamon Bay	28.3507 N	80.6129 W	134.8 ± 40	3599.8 ± 524.3	132.6 ± 21.8	188.1 ± 2.2	0.2
DBB-10	08/26/22	Diamon Bay	28.3507 N	80.6129 W	167.6 ± 48.5	4063.5 ± 174.8	109.0 ± 44.2	169.1 ± 20.0	0.3
DBB-11	08/26/22	Diamon Bay	28.3507 N	80.6129 W	119.2 ± 55.4	3139.2 ± 693.9	70.0 ± 18.2	167.7 ± 33.1	0.3
DBB-8	08/26/22	Diamon Bay	28.3507 N	80.6129 W	92.7 ± 34.3	2779.6 ± 154.3	67.4 ± 25.8	153.0 ± 137.3	0.4
DB-7	05/26/22	Diamond Bay	28.3507 N	80.6129 W	153.2 ± 38.3	3504.2 ± 803	81.5 ± 30.9	129.8 ± 40.8	0.4
DB-9	05/26/22	Diamond Bay	28.3507 N	80.6129 W	145.5 ± 71	3274.9 ± 187.6	117.9 ± 24.7	173.0 ± 18.5	0.2
DB-5	05/26/22	Diamond Bay	28.3507 N	80.6129 W	123.6 ± 88.5	2635.9 ± 989.7	96.9 ± 59.7	163.6 ± 20.5	0.5
AVG					133.8 ± 24.7	3285.3 ± 491.8	96.5 ± 24.8	163.5 ± 18.2	0.3 ± 0.08
ST18	06/15/22	Station 1 (ST)	28.4202 N	80.6452 W	118.1 ± 44.2	3396.2 ± 341.9	84.1 ± 25.0	100.7 ± 16.6	0.4
ST-13	06/15/22	Station 1 (ST)	28.4202 N	80.6452 W	179.2 ± 37	2965.2 ± 362.2	85.4 ± 23.8	119.4 ± 24.2	0.4
ST-9	06/15/22	Station 1 (ST)	28.4202 N	80.6452 W	72.4 ± 57.2	2475.8 ± 925	46.7 ± 24.2	102.1 ± 9.5	0.5
ST-4	06/15/22	Station 1 (ST)	28.4202 N	80.6452 W	53.0 ± 32.5	2239.9 ± 715.3	39.5 ± 11.7	81.9 ± 8.8	0.5
STB-14	07/20/22	Station 1 (STB)	28.4202 N	80.6452 W	105.8 ± 67	2633.7 ± 597	105.4 ± 35.5	140.4 ± 49.8	0.4
STB-15	07/20/22	Station 1 (STB)	28.4202 N	80.6452 W	103.6 ± 26.6	2558.7 ± 299.4	110.8 ± 18.9	163.6 ± 41.2	0.4
STB-23	07/20/22	Station 1 (STB)	28.4202 N	80.6452 W	169.7 ± 70.0	2601.4 ± 353.8	101.0 ± 15.2	192.3 ± 32.8	0.3
STB-12	07/20/22	Station 1 (STB)	28.4202 N	80.6452 W	177.9 ± 50.0	4167.6 ± 522.4	120.4 ± 43.7	147.4 ± 40.6	0.4
AVG					122.5 ± 48.6	2879.8 ± 626.35	86.7 ± 29.5	131.0 ± 36.8	0.4 ± 0.08
PFB-17	07/20/22	Platform (PFB)	28.3670 N	80.6331 W	139.1 ± 54	3576.2 ± 946	87.7 ± 45.3	74.3 ± 27.3	0.5
PFB-6	07/20/22	Platform (PFB)	28.3670 N	80.6331 W	184.3 ± 19.6	4911.1 ± 1046.7	83.2 ± 14.5	100.1 ± 16.6	0.5
PFB-19	07/20/22	Platform (PFB)	28.3670 N	80.6331 W	205.8 ± 68.8	3031.5 ± 571.2	71.6 ± 23.7	151.2 ± 36.6	0.3
PFB-20	07/20/22	Platform (PFB)	28.3670 N	80.6331 W	168.5 ± 42	3122.5 ± 296	74.8 ± 33.3	103.9 ± 26.5	0.3
PF-8	06/15/22	Platform (PF)	28.3670 N	80.6331 W	136.0 ± 49	2950.8 ± 540	69.3 ± 35.9	60.1 ± 21.0	0.4
PF-13	06/15/22	Platform (PF)	28.3670 N	80.6331 W	131.8 ± 50	3523.8 ± 945.2	95.1 ± 50.6	33.1 ± 6.1	0.7
PF-9	06/15/22	Platform (PF)	28.3670 N	80.6331 W	153.7 ± 43	2886.9 ± 83	83.7 ± 11.6	91.4 ± 16.2	0.6
AVG					159.9 ± 27.7	3429.0 ± 707.3	80.8 ± 9.3	87.7 ± 37.4	0.5 ± 0.16
B42-15	05/26/22	Beacon 42	28.7562 N	80.7661 W	63.2 ± 28.6	2877.0 ± 950	65.7 ± 65.3	241.1 ± 69.6	0.4
B42-1	05/26/22	Beacon 42	28.7562 N	80.7661 W	56.2 ± 9.0	3580.9 ± 896	64.8 ± 4.0	95.6 ± 15.6	0.5
B42-32	05/26/22	Beacon 42	28.7562 N	80.7661 W	122.7 ± 22.4	2591.7 ± 215	61.1 ± 10.1	181.5 ± 37.3	0.5
B42-33	05/26/22	Beacon 42	28.7562 N	80.7661 W	121.9 ± 40.3	2105.6 ± 1085.8	81.1 ± 37.2	60.8 ± 6.6	0.6
B42B-7	08/26/22	Beacon 42 (B42B)	28.7562 N	80.7661 W	138.9 ± 20	2405.6 ± 281.7	88.1 ± 15.0	103.1 ± 6.9	0.3
B42B-2	08/26/22	Beacon 42 (B42B)	28.7562 N	80.7661 W	138.9 ± 57.4	1385.2 ± 77.9	74.9 ± 43.1	78.4 ± 19.7	0.3
B42B-10	08/26/22	Beacon 42 (B42B)	28.7562 N	80.7661 W	173.5 ± 40.9	1744.1 ± 697	80.5 ± 2.8	148.7 ± 29.9	0.4
AVG					116.5 ± 42.4	2384.3 ± 730.7	73.7 ± 10.1	129.9 ± 64.1	0.4 ± 0.1
BR17-23	07/07/17	Banana River (BR)	28.3451 N	80.6528 W	42.6 ± 30.5	4692.7 ± 1504.8	79.2 ± 60.6	254.2 ± 60.9	0.2
BR17-21	07/07/17	Banana River (BR)	28.3451 N	80.6528 W	154.3 ± 38	3544.8 ± 444	57.9 ± 11.0	257.5 ± 52.4	0.1
BR17-16	07/07/17	Banana River (BR)	28.3451 N	80.6528 W	318.4 ± 53.8	4516.2 ± 1738.1	85.3 ± 29.8	401.8 ± 111.0	0.2
AVG					171.8 ± 138.7	4251.2 ± 618.2	74.1 ± 14.4	304.5 ± 84.2	0.2 ± 0.02

**Table 2:** List of primers used in this study.

Primer	Sequence (5′-3′)	Source	Notes
PyroRq	GCCGCCGCCACCATATCC	[38]	sxtA4 Qpcr/qRT-PCR
sxt072	CTTGCCCGCCATATGTGCTT	[39]	
rbcL186F	TGGCGAGGCCTGTTACGC	[38]	rbcL qPCR/qRT-PCR
rbcL312R	CGAGCCGGTCTCCTTGATG		
Lcf54F	AGGCCATCGAGGCGGGC	this study	qPCR
Lcf154R	CAGTGCAGACATCGCCAAAC	this study	

## Data Availability

Data is contained within the article or Supplementary Material.

## Supplementary Materials

The following supporting information can be downloaded at: https://www.mdpi.com/article/10.3390/toxins18100423/s1, Figure S1: Correlation plot of *sxtA4* gene copies per cell and toxin quota per cell from 15 field samples collected at DB and TM over the course of the 2024 season; Figure S2. Melt Curve Plot of *Pyrodinium* isolates. All samples yielded a single Tm of 86.8–87; Figure S3. *Lcf* standard curve.

## Abbreviations

The following abbreviations are used in this manuscript:

STX	Saxitoxin
NIRL	Northern Indian River Lagoon
IRL	Indian River Lagoon
CNV	Copy Number Variation
HAB	Harmful Algal Bloom
PSP	Paralytic Shellfish Poisoning
SPFP	Saxitoxin Pufferfish Poisoning
DB/DBB	Diamond Bay
TM	Titusville Marina
ST/ST1	Station 1
PF/PFB	Platform
B42	Beacon 42
BR	Banana River
