# Supplementary material for: Toxin Dynamics and Genomic Diversity in Pyrodinium bahamense Populations from the Indian River Lagoon, Florida
Source: Toxins (Basel). 2026 Sep 30;18(10):423. doi: 10.3390/toxins18100423 (PMC13709659; doi:10.3390/toxins18100423)
Supplement: Supplementary file 1 [file toxins-18-00423-s001.zip › toxins-4326926-supplementary.pdf]

toxins

Supplementary Materials: Toxin Dynamics and Genomic Diversity in *Pyrodinium bahamense* Populations from the Indian River Lagoon, Florida

Kathleen Cusick, Jackie Palau, Malcolm N. McFarland and Edith A. Widder

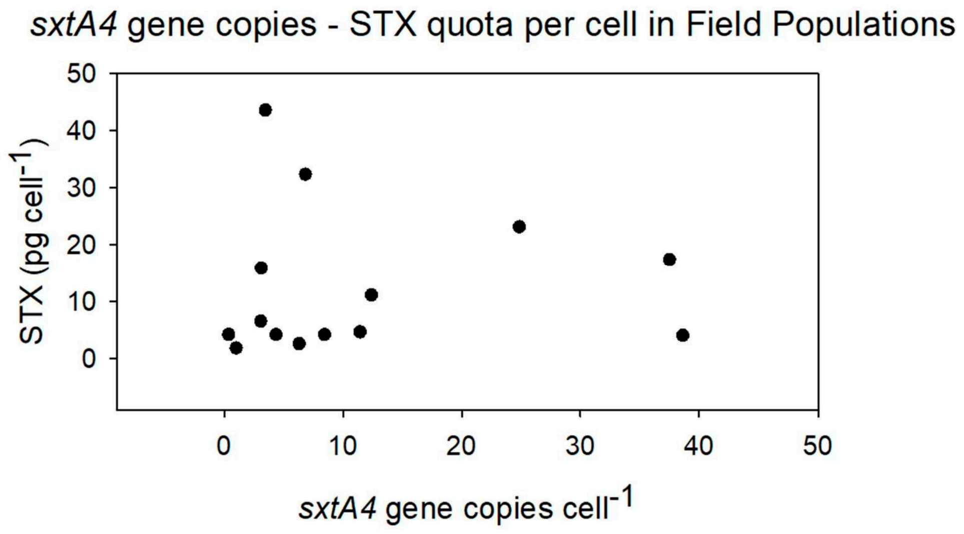

Figure S1. Correlation plot of *sxtA4* gene copies per cell and toxin quota per cell from 15 field samples collected at DB and TM over the course of the 2024 season.

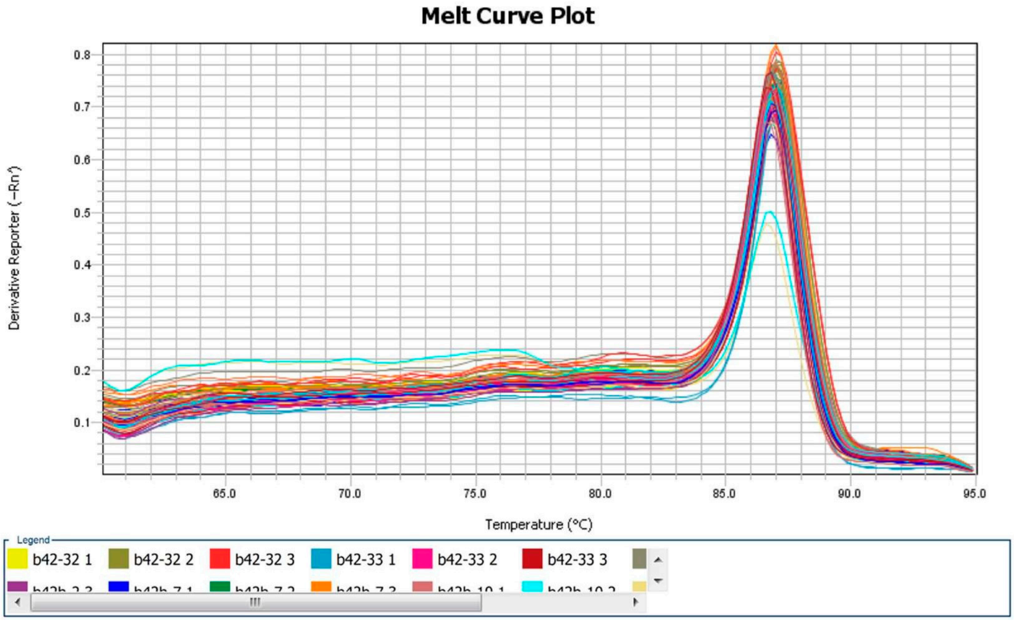

Figure S2. Melt Curve Plot of *Pyrodinium* isolates. All samples yielded a single Tm of 86.8-87. .

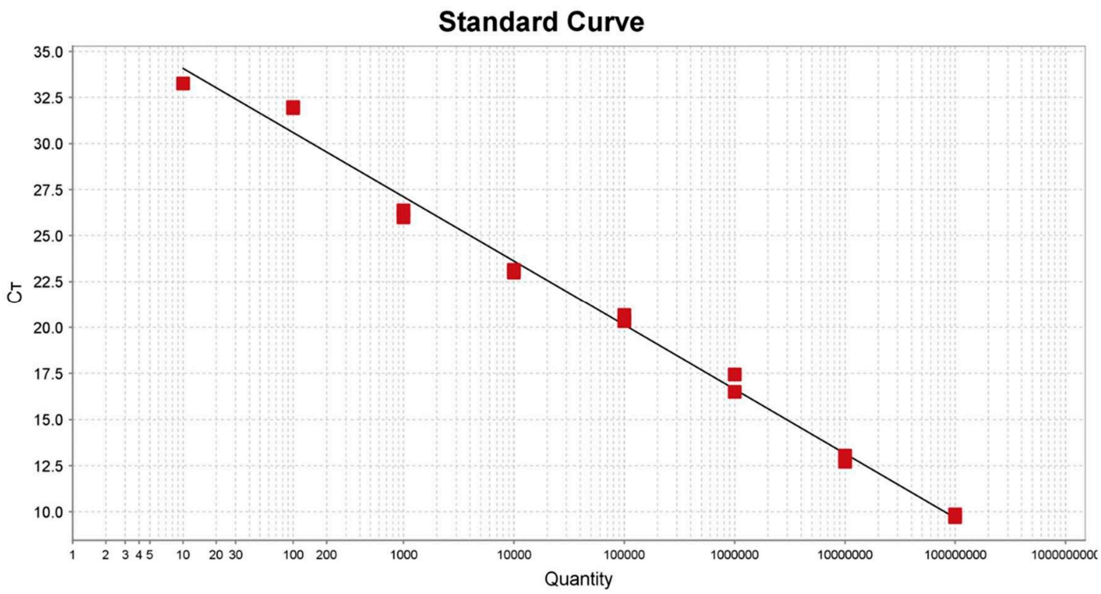

Figure S3. *Lcf* standard curve.
